# A Timely Referral to Palliative Care Team Improves Quality of Life

**DOI:** 10.4103/0973-1075.76233

**Published:** 2011-01

**Authors:** P Saraswathi Devi

**Affiliations:** Department of Anaesthesiology and Pain Relief, Kidwai Memorial Institute of Oncology, Bangalore, India

**Keywords:** Palliative care, Team approach, Early referral, Quality of life

## Abstract

In the trajectory of disease progress and treatment plan, patients and the family members are confronted with challenging situations like unsurmountable physical distress, inadequate coping patterns, unanswered spiritual issues in the background of serious threat to very existence of life leads to a debilitating Quality of life.The Palliative Care team approach addresses all the issues and also sees the patient to go through the protocols of Palliative care management as well as Oncological treatment plan. Further, this fecilitates a smooth transition from the hospital to home and hospice care. Various studies conducted globally revealed that patients received palliative care intervention along with oncological treatments had higher scores of Quality of life compared to patients received onlyoncology care alone.This article discusses the various factors contributing to late referrals to palliative care team and also care giver’s views pertaining to need for early referral. Timely referral to palliative care minimises the patient’s and care giver’s distress, ensures modest Quality of life and appropriate measures at the end of life care.

## INTRODUCTION

Alexander Graham Bell once said “Great discoveries and improvement invariably involve the co-operation of many minds.” Though he was referring to philanthropy, this statement can be applied to the spirit of teamwork in palliative care.

Dealing with symptoms of any painful or serious or life limiting illness is a challenging task for any health care professional. Palliative care provides an answer and has a vital role to play in the management of such of those patients whose very existence is under a serious threat, which may well be compounded by pain and other distressing symptoms. Palliative care is a comprehensive care, tailored to cater to the individual patients’ needs and works in synergy with the primary treatment the patient is receiving. In a disease like cancer, in spite of great advancements in therapy and extensive research, patients still have to face a lot of problems such as uncertainty in prognosis, intolerable symptoms [as a result of both the disease and its treatment] which may leave him/her with disabilities, high levels of anxiety and/or depression which is sure to undermine his/her self-confidence–in short, the quality of life is at its lowest ebb. It is here that palliative care professionals can chip in, not only to provide relief from physical symptoms, but also to give them psychosocial support, which can help in blunting the impact of the disease and see the patient through the treatment protocols.

Palliative care provides emotional support not only to the patient, but also to his family and helps in opening up discussions about disease-related treatment choices and management of symptoms related to it.

Palliative care helps the patient and the family to communicate better with each other and with health care professionals. It helps them identify the priorities and set goals for the future that can lead to a meaningful, satisfactory life for both the patient and themselves. Studies reveal that palliative care ensures care more in line with patients wishes and meets the emotional and spiritual needs of the patient.[1] The needs and the distress caused by the disease are better understood by the palliative care team members as they are able to spend time, and their services can be utilized both during cancer treatment and also at the end of life.

Team approach makes sure that the patient experiences a smooth transition from the hospital to other services such as home care or nursing facilities. This results in a well planned complete treatment for all the symptoms throughout the illness–treatment that takes care of the present condition and anticipates the future needs.

Palliative care is provided by a team of specialists that includes the palliative care physician, palliative care nurse, social worker, pharmacist, nutritionist, counselor, physiotherapist, spiritual care giver, and volunteers. To provide a reasonable and meaningful quality of life (QOL) is the central theme of palliative care. The domains included in health-related QOL are physical function, symptom distress, family well being, emotional well being, social function, and spiritual satisfaction. The other areas that could be focused on are the financial impact of disease, sexual function, body image concerns, and spirituality crisis.

QOL depends on the site of cancer and its importance in head and neck cancers are widely studied. Balman described QOL in inverse relationship to the size of the gap between the individual’s expectations and the reality of the situation; the smaller the gap the better the QOL. This gap principle is also implicitly present in most QOL questionnaires, and thus the score reflects QOL relative to the individual’s expectations. Thus palliative care focuses on physical comfort, alleviation of symptom distress as well as social and emotional needs-all essential components of QOL. This is offered both during cancer treatment and at the end of life “Early referrals to palliative care team are essential not only to relieve the symptom distress but also to improve treatment outcomes and QOL for people with cancer” says Tatsuya Morita, MD, Department of Palliative and Supportive Care, Palliative Care Team, Scirei Hospice in Shizuoka, Japan, and lead author of the study. “Patients and their families should feel comfortable discussing end of life care with their physicians and each other so that palliative care services can be given at the most appropriate moment for the patient.”[2]

Patients with advanced lung cancer who received integrated palliative care early on during treatment had a better QOL and survived for two months longer (11.6 months versus 8.9 months) compared to patients receiving standard care (chemotherapy) according to a study published in the August 19, 2010 issue of New England Journal of Medicine. This study was conducted by a team of clinicians in Boston, designed by Jennifer Temel a lung cancer Oncologist at Massachusetts, a randomized trial on 151 patients with metastatic lung cancer.[3]

Another lengthy Randomized controlled study conducted at Lebanon on 322 patients newly diagnosed with advanced cancer from November 2003 through May 2008 revealed that the patients received palliative care interventions along with oncology care had higher scores of QOL and mood compared to the patients received only oncology care.[4] Indeed, early referral to palliative care not only facilitates timely diagnosis and treatment of symptoms, but also minimizes care giver distress and aggressive measures at the end of life.[5]

Despite this, misconception about palliative care still exist in developed and in most developing countries-even among the medical professionals-that palliative care indicates that treatment has failed-that it means giving up-that patients who are receiving or attending to palliative care facilities are considered “TERMINAL.” This leaves the patient and the family confused and often unwilling to receive the care and would often resist the offer of the team to visiting the patient at home. The services of the home care team is, however, slowly gaining acceptance albeit in only the advanced stages.

Referrals to palliative care often come too late to improve QOL for patients with cancer. The referral of patients to palliative care team occurs late in the trajectory of illness at an average of 30-60 days before death.[6] A new study carried out in Japan, on family members of people who have died of cancer found that nearly half of respondents believed that referrals to palliative care were given late in the course of the illness.[7]

Doctors find it difficult to make early referrals to palliative care for many reasons. These include being unsure of the disease process, possibility of periods of remission, inadequate communication skill, lack of knowledge about palliative care, lack of support, time, and lack of team accessibility.[8]

Dr. Temel’s study was discussed extensively and agreed upon at American society for clinical oncology annual convention 2010, that getting the palliative care team (entire team of palliative care specialists) involved early in the care of cancer patients definitely improves QOL and symptom control.[9]

Some recent comments from families of patients who died without being referred to palliative care teams or referred late.

- Much of the misery could have been lessened had someone been there to address the physical and emotional aspects of the disease from the very diagnosis of the disease. It seems logical that less pain and stress would have led to improved QOL
- Early integrated palliative care could have helped patients live longer.[10]
- Majority of families preferred an earlier consultation.^5^

A study conducted at several sites in Australia regarding late referral or non-referral to palliative care for people with life-limiting disease states that lack of knowledge about palliative care services and its benefits was the over-riding reason among all health care providers, patients and families.[11]

## CONCLUSION

There is no doubt that the QOL of a patient can be enhanced greatly by incorporating palliative care early in the course of the illness, perhaps even from the day of the diagnosis. Oncologists and primary care physicians must be sensitized to the role of palliative care team and must be encouraged to take them as partners early in the disease if they are to give a comprehensive and total care to the patients, to help them go through the treatment and to support them till the very end.

“Death and dying is never an easy business. However, we do have tools to help ease this part of life. It is our job as health care professionals to use them fully and to use them promptly. Now we have no excuse.”
